# Maze-solving in a plasma system based on functional analogies to reinforcement-learning model

**DOI:** 10.1371/journal.pone.0300842

**Published:** 2024-04-10

**Authors:** Osamu Sakai, Toshifusa Karasaki, Tsuyohito Ito, Tomoyuki Murakami, Manabu Tanaka, Makoto Kambara, Satoshi Hirayama

**Affiliations:** 1 Department of Electronic Systems Engineering, The University of Shiga Prefecture, Hikone, Shiga, Japan; 2 Regional ICT Research Center for Human, Industry and Future, The University of Shiga Prefecture, Hikone, Shiga, Japan; 3 Department of Advanced Materials Science, The University of Tokyo, Kashiwa, Chiba, Japan; 4 Department of Systems Design Engineering, Seikei University, Musashino, Tokyo, Japan; 5 Department of Chemical Engineering, Kyushu University, Fukuoka, Japan; 6 Department of Materials and Manufacturing Science, Osaka University, Suita, Osaka, Japan; Tokai University, JAPAN

## Abstract

Maze-solving is a classical mathematical task, and is recently analogously achieved using various eccentric media and devices, such as living tissues, chemotaxis, and memristors. Plasma generated in a labyrinth of narrow channels can also play a role as a route finder to the exit. In this study, we experimentally observe the function of maze-route findings in a plasma system based on a mixed discharge scheme of direct-current (DC) volume mode and alternative-current (AC) surface dielectric-barrier discharge, and computationally generalize this function in a reinforcement-learning model. In our plasma system, we install two electrodes at the entry and the exit in a square lattice configuration of narrow channels whose cross section is 1×1 mm^2^ with the total length around ten centimeters. Visible emissions in low-pressure Ar gas are observed after plasma ignition, and the plasma starting from a given entry location reaches the exit as the discharge voltage increases, whose route converging level is quantified by Shannon entropy. A similar short-path route is reproduced in a reinforcement-learning model in which electric potentials through the discharge voltage is replaced by rewards with positive and negative sign or polarity. The model is not rigorous numerical representation of plasma simulation, but it shares common points with the experiments along with a rough sketch of underlying processes (charges in experiments and rewards in modelling). This finding indicates that a plasma-channel network works in an analog computing function similar to a reinforcement-learning algorithm slightly modified in this study.

## Introduction

Maze-solving is a task that fascinates not only general people solving puzzles but also scientists in a wide range of categories [1]. Its scientific quest being started as a mathematical problem, it is a benchmark of the intelligent level for various media such as living tissues [2], chemical systems [3], fluid flow [4], and ensembles of electric devices (like memristors) [5], most of which possess nonlinear properties in their individual element and a complex network in their unity. The task of maze-solving includes a problem of non-deterministic polynomial-time (NP problem) [6] in the comprehension in computational studies of complexity, and such nonlinear elements and their networking are functional for the solution in the previous studies listed above. Maze-solving is also a gateway to other physical computing schemes like digital logic circuits and analog computers [4].

Discharge plasma possesses nonlinearity as well, and several experimental studies for maze-solving have been reported so far. In a glow discharge, electrons and ions actively move at random with neutral particles according to principles on electric-field drift and particle diffusion via spatial density gradient [7]. General glow-discharge experiments in a rare-gas space seem to be quite simple and easy to reproduce primitively if the implementation of a vacuum pump and gas supply system with sufficient high-voltage electric power supply are available, and the entire phenomenon is completed remarkably in a short time. In one experiment [8], microchip structure with tiny trenches was etched on a dielectric plate to configure a maze, and high-voltage (1–6 kV) direct-current power supply generated glow-discharge plasma between electrodes located at starting and ending points. Visible plasma emission displayed the shortest path successfully, and such high voltage would have been required to accelerate initial electrons directly between both ends. Another previous experiment about plasma ignition in a multi-wall low-pressure space also testified that maze-solving was successful as a path along glow-discharge plasma [9]. According to the report of this experimental study, the visible image of the glow discharge showed a striation pattern [7], which is an evidence of importance in electric potential profiles to form a plasma path in the corresponding experimental apparatus. The authors also performed an experiment of surface microdischarges [10], and this maze-solving method was applied to biomaterial processing [11], which is a technical application of this plasma maze-solving method.

We also pay attention to reinforcement learning (RL) [12–16], which offers an effective computing algorithmic model for maze-solving [14, 15, 17, 18]. RL typically works well for problems that includes close interactions between agents and their environment, and favorable next action is selected to optimize the final goal in a given problem. Although a simple route-finding action induces no change on the environment which is given as an exit location of the maze, RL is still powerful for finding the shortest route among the choices in a complicated labyrinth. As a maze solver, the heuristic property that RL substantially possesses is beneficial, and its feasibility can be upgraded in several ways. For instance, after the initial proposal of Q-learning [12], inclusion of multi-agent models and its decentralization system was recently proposed [19], which enhances rapid convergence with robustness. In the case of maze-solving in a simple RL model, the distribution of the reward spreading from the exit is a key issue, and its spatial pattern is configured in automatic and arbitrary iterations. The pattern created after many iterations are similar to physical diffusion profiles of particles from the source, as shown later in this report.

An agent action questing the maximum reward is optimized in a RL model, while particle motions in plasma are regulated along the physical principles. In this study, we compare these two dynamic systems to search for plasma maze-solving functions which seem to be intelligent and also to reveal RL functions as an analogous simulator for some physical events. The configuration for plasma generation is different from the previous study [9] and close to the other [8]; we designed a fine channel structure surrounded by dielectric walls whose surfaces are almost at a floating potential [20], and plasma particles sense the electric potential not only governed by electrodes but depending mainly on charged dielectric in dielectric barrier discharges [21, 22]. This electric charging is a result of charged particle accumulation in the preceding bipolar discharge pulses. After demonstrating route findings by visible plasma channels, we compare the experimental results with the RL calculations in the model similar to the maze structure used in the experiments. Here, we follow achievements by the Q-learning model [12, 13] in which reward is distributed from the maze exit by a large number of accumulated iterations, and modify it to imitate motions of a huge number of plasma particles mobile in the experimental space.

## Methods

### Experimental apparatus

Experiments were performed in a system composed of a small vacuum chamber with a carved dielectric pattern, a vacuum pump with gas feeding facility, and an electric power supply. Most of them are typical in experiments of plasma generation, and carved maze patterns on the surface of the dielectric plates induces a distinguishable perspective for experimental performance in our study. Following our previous brief report [20], here we demonstrate various experimental results to reveal functions for maze-solving.

A conventional chamber for plasma generation forms a discharge space that occupies the most of inner volume, and inserted metallic electrodes connected to the outer electric power supply generates plasma through ionization in an intensified electric field [23]. Dielectric components are used as supporters for spatial arrangements of metallic parts. The gas feeding equipment controls the pressure in the discharge space together with multiple valves; for instance, when the electrode gap is several tens of mm, the gas pressure is at tens of Pa in a typical plasma experimental setup [7].

**Fig 1:**
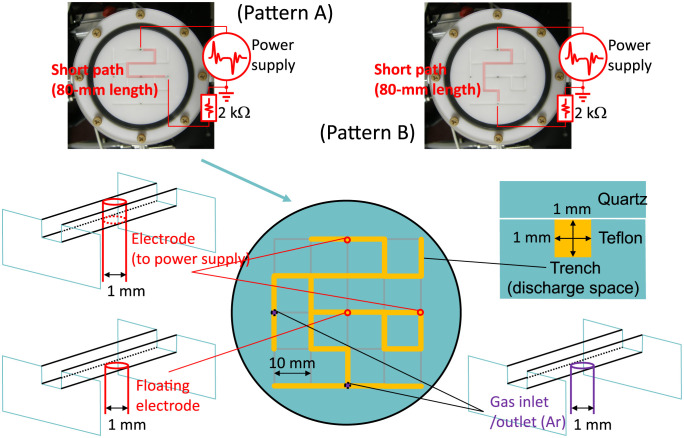
Spatial configurations of experimental setup. Plasma channels created in Ar gas by 20-kHz high-voltage power supply are in trenches on the surface of a Teflon plate. Electrodes of stainless steel are for electric voltage imposition and floating potential detection. Details shown here is for pattern A, whereas those for pattern B is depicted in S1 Appendix.

However, in our chamber system (in Fig 1), all of the discharge space consisted of microchannels whose square cross section was 1 by 1 mm in size as a trench of a Teflon dielectric plate. Channels were arranged in a square lattice configuration, and the lattice constant was 10 mm. Microchannels were open or closed by carving them on the dielectric surface according to each maze design. Thus, a microchannel was almost independent from each other without cross talks of electrical potentials, unlike the previously study in which channels were adjacent across a thin dielectric wall [9]. Electrodes, which were 1-mm-diemater circular poles of stainless steel, can be located at grid crossing points of the lattice, and the high-voltage electrode works as the entry or starting location of the maze, while the grounded electrode corresponds to the exit, goal or finishing location. In the following experiments, we fixed the dielectric carving pattern and the entry node, while the goal node was set to different locations in two experiments (patterns A and B). The top plate of the chamber was also a dielectric material, quartz glass, to complete dielectric channels and to make the plasma image visible from the outside.

The output of our electric power supply (Haiden Laboratory PKF-2KW) was in a bipolar pulsed shape, with small ringing subsequent waves. This output waveform, different from a simple sinusoidal waveform, is not dominant for the function of maze-solving, but useful to observe residual potentials or remaining charges on the dielectric after the main pulses. Bipolar pulses are essential for our system to induce charged dielectric; after imposing a positive pulse on a high-voltage electrode, electrons flow into it, and the dielectric surface in the microchannels around it are charged up negatively. Next, when the negative pulse comes, the charged dielectric works to enhance its negative potential, and the charge was replaced by the positive one after a plasma shot. Plasma was generated in Ar gas with small gas flow (≤30 sccm) from the inlet to the outlet holes through all the microchannel structure; gas pressure was higher than 100 Pa. This relatively high gas pressure in comparison with conventional plasma experiments is attributed to the small cross section in this experimental setup [24], and this plasma scheme with highly frequent collisions among particles is maintained by surface dielectric barrier discharges which can be operated at high gas pressure [21, 22].

Experimental data are acquired as visible images taken by a CCD (Charge Coupled Devices) device by a widely-available digital camera (Olympus SP-550UZ) and time-evolution signals recorded by a conventional digital oscilloscope (Tektronix TBS1064) with a high-voltage probe (Tektronix P5100). Raw datasets acquired in the experiments are displayed in S1 Appendix.

### Reinforcement learning model

Our RL model is categorized in typical Q-learning methods [12], except the following points: rewards possess positive and negative polarities, corresponding electric potentials externally applied by electrodes in one voltage pulse in the experiments, and initially-set rewards *r* are transported by random virtual motions for the calculation of expected discounted reward *Q*. The model structure in which an agent is mobile is a conventional network or graph [25], where nodes correspond to the grid crossing points and edges, with slightly negative reward, exist if the corresponding microchannels are open as maze paths in a given carving pattern. The agent moves from the entry toward the goal node, and selects a path with larger *Q* value in the direction of positive polarity, which is the expected discounted reward for executing action *a* at state *s* [12]. We note that *Q* and *r* are given on both directions with different quantities on one edge. With initial sources of the reward *r* located near the goal positions, *Q* has been distributed from the adjacent edges with parameters of the discount factor *γ* and the learning rate *α* in a typical RL formula, given as:

$$\begin{array}{c} Q_{i}\left(s_{j},a_{j}\left(s_{j}\right)\right)=\left(1-\alpha \right)Q_{i-1}\left(s_{j},a_{j}\left(s_{j}\right)\right)\\ +\alpha [r_{j}+\gamma \underset{a_{j+1}\left(s_{j+1}\right)}{\text{max}}Q_{i-1}\left(s_{j+1},a_{j+1}\left(s_{j+1}\right)\right)], \end{array}$$ (1)

where *j* indicates the number of episodes (or real agent motions) calculated so far. Beware that a local state (or a local position which now the agent possibly stays at) *s* is randomly chosen at the hypothetical iterations *i* (10,000 times) of *Q*-transport calculation by Eq (1), even if the agent never stays there, for derivation of the *Q* value distribution at each episode. Then, the *Q* value is updated to be the saturated state before the actual action of the agent *a*. The coeffecient ratio between two terms on the right hand side ((1 − *α*) : *α*) of Eq (1) means that learning is performed in the rate of *α* from the initial and future rewards, while in the remaining portion (1 − *α*) *Q* follows the last value without updates. By *γ*, *Q* increases by selecting the next action at *j* + 1 in a rule which is here the choice of the maximum value (“max”).

**Fig 2:**
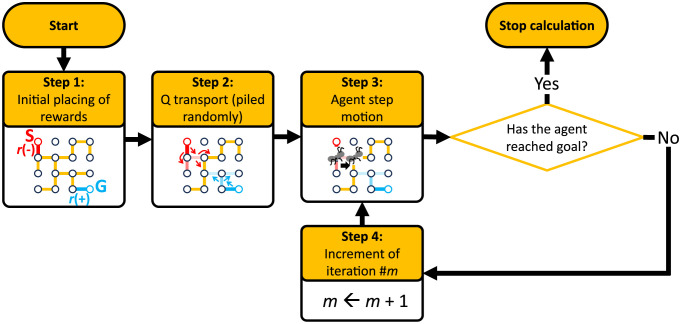
Workflow for route finding based on RL model.

In our model which is shown in Fig 2, we modify Eq (1) as follows:

$$\begin{array}{c} Q_{i}\left(s_{j},a_{j}\left(s_{j}\right)\right)=\left(1-\alpha \right)Q_{i-1}\left(s_{j},a_{j}\left(s_{j}\right)\right)\\ +\alpha [r_{j}+\gamma \;\underset{a_{j+1}\left(s_{j+1}\right)}{\text{random}}\;Q_{i-1}\left(s_{j+1},a_{j+1}\left(s_{j+1}\right)\right)]. \end{array}$$ (2)

Here, the mathematical function defined tentatively, “random,” indicates random selection among the possible options with equiprobability. By replacing “max” by “random,” the reward distribution is performed randomly, which is quite similar to particle motions in the thermodynamical diffusion process.

We set the initial placing of rewards as follows: if one end of an edge is the goal or the entry node of the maze, the edge has initial rewards with a positive amount (e.g., +100) in the inlet direction or a negative one (e.g., −100) in the outlet direction, respectively, represented by *r* in Eq (1). *r* on the major or general edges is set to be −10. Then, by spatial transport of *Q* by accumulation of random hypothetical individual motions in Eq (1), a *Q* profile is calculated.

After this initial calculation of *Q*, the agent actually moves to find the next path along one edge with the largest *Q*. Since this agent motion does not change the environment of the RL model like *r* locations, unlike typical RL calculation steps, we can skip the updating procedure of the *Q*-profile calculation at every iteration step. The agent iteratively moves to the next node by selecting the path with the largest *Q* at each current node until arrival at the goal node. Finally, we step to confirm the consistency of the route with the shortest one.

Computation in this study was performed by a commercially-available 64-bit personal computer by a homemade Python code; the details are described in S1 Appendix. One calculation for searching for one route is completed within one second.

## Results

### Experiments: Route evolution quantified by ambiguity reduction

**Fig 3:**
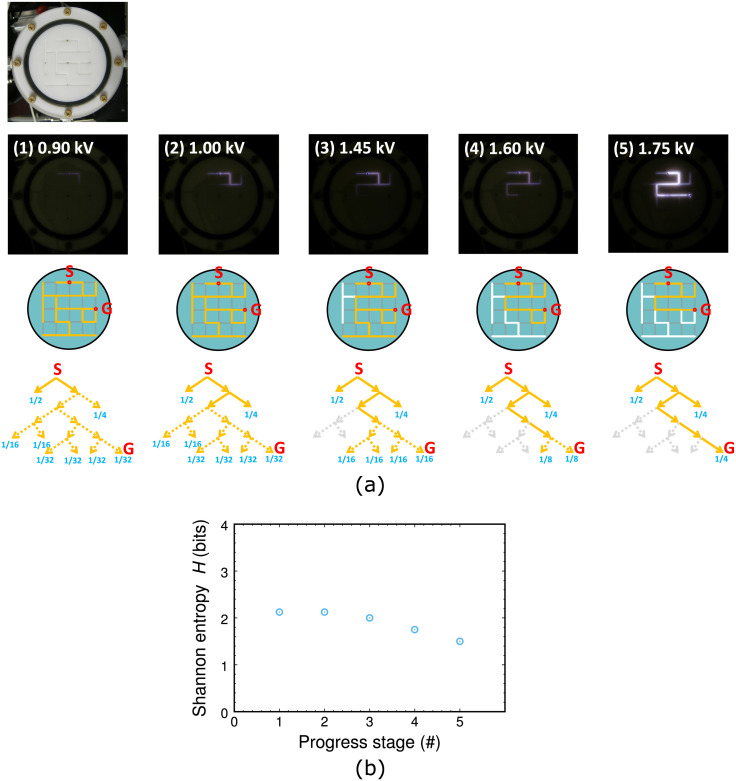
(a) Visible plasma images and corresponding path processions with branch probabilities in case of pattern A depicted in Fig 1, with Ar gas pressure 300 Pa. Voltage depicted is at peaks of bipolar pulses. At each stage, possible paths selection with equiprobability are exemplified in attached directed graphs, where solid yellow paths are in the fixed route, hatched yellow paths are in possible routes, and white paths are removed from the route. (b) Variation of Shannon entropy as the route is found in (a). Numbers of progress stages are listed in images in (a).

Fig 3(a) shows visible plasma images at several levels of the discharge voltage. The ignition started from the high-voltage electrode, and after imposition of the sufficient voltage, the plasma channel reached the goal or the exit of the maze. As the plasma channel increased its length, it spread in multiple directions at a node where more than three edges are connected, although the shortest route leading to the goal was always included. Then, when it reached the goal, the emission along the route became intense, finishing its quest.

Fig 3 also displays ambiguity reduction as the route converged. Here we calculate Shannon entropy *H* [26] from probability distributions in path selection, as displayed in Fig 3(b). If a route finder works randomly, a forward branch is selected with equiprobability. Then, at each branch, the probabilities are assigned equally, and a dead end becomes a leaf or termination node of the graph tree that is given a probability *p*_*k*_, where the summation of *k* means the number of the leaf nodes. As the route proceeds to the goal, some edges connecting to each branch are not selected, their probabilities turning into zero. This converging process of the route can be estimated using by *H* in the unit of bits, simply given as:

$$\begin{array}{c} H=-\sum_{k}p_{k}\;{\text{log}}_{2}p_{k},\;\;\;\sum_{k}p_{k}=1. \end{array}$$ (3)

and *H* represents an ambiguity level of states in the system. Generally, more leaf nodes exist, a larger value *H* possesses since the level of ambiguity in branch selection is higher. On the other hand, if there is no other choice to select a path to goal along a route with no possible branches, *H* is zero, the minimum value.

In Fig 3, as the plasma channel traces the correct route, paths removed from the route (in white) increasing, and consequently branches decreases along the route which converged into a simple line (changing from hatched to colored edges). Finally, the route finding was completed when the plasma channel reached the goal node or the ground electrode. This evolution is quantified by decrement of *H*; while the route was not fixed, *H* stayed at a high level, but it decreased after the converging process went on. Some paths out of the route with plasma emission remained when the route finding was completed, with a finite *H* value. If such unnecessary edges were successfully removed, *H* would drop down to zero.

**Fig 4:**
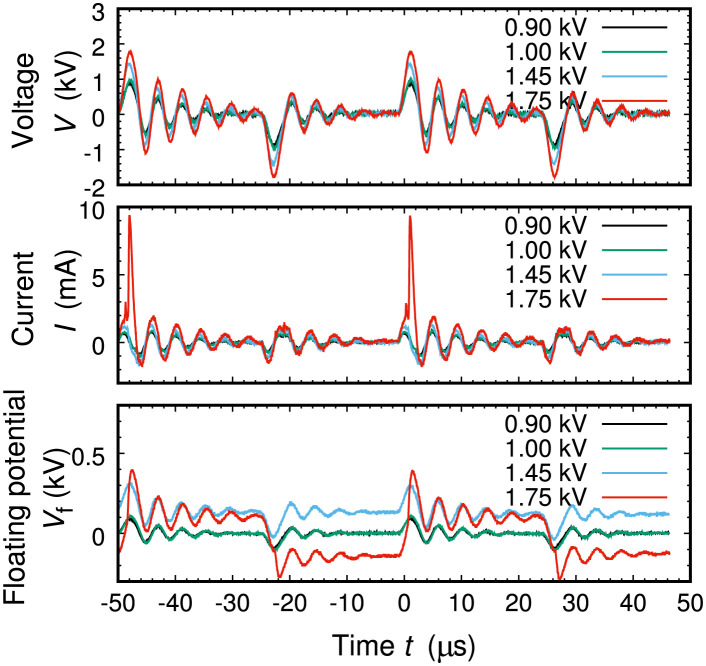
Time evolutions of applied voltage from power supply, flowing discharge current, and floating potential in case of pattern A depicted in Fig 1. Parameters shown as inset numerals in voltage correspond to the bipolar peak voltage, with Ar gas pressure 300 Pa.

To generate plasma channels shown in Fig 3(a), bipolar pulse voltage by the power supply was applied between two electrodes, and discharge current was monitored by detecting the voltage drop across the series resistance at 2 kΩ (Fig 4). We note that the current signal includes the component of displacement current in its large portion, and an additional calculation step yields the net transport of charged particles, which will be discussed using Fig 6. To understand complex behaviors observed in the signal of the floating potential, further consideration is also required with additional analysis.

### Experiments: Parameter range for plasma route findings

This plasma generation was successful in a wide range of gas pressure, as described below. In a typical glow discharge, at either direct-current or high-frequency operations of discharge voltages, the optimum gas pressure exists for one electrode gap [7]. Apart from the point with the lowest discharge voltage, the voltage increases sharply at both higher and lower pressures since electrons run with collisions in the parallel electrode gap. Consequently, the pressure range for operation is limited around the optimum gas pressure (around 100 Pa) for several tens of mm gap. However, electrons in the microchannel, bending at several branches, cannot run smoothly between electrodes, and strongly influenced by surface potentials on the dielectric walls. Their mobility had not been rigorously analyzed so far, but they contain similar motions in dielectric barrier discharges [21, 22] in which the transport of charged particles seems to be frequent between dielectric surfaces with charged particles in opposite signs. Then, flight distance of charged particles vary within the microchannel length, from ∼10 mm to ∼100 mm in our case.

**Fig 5:**
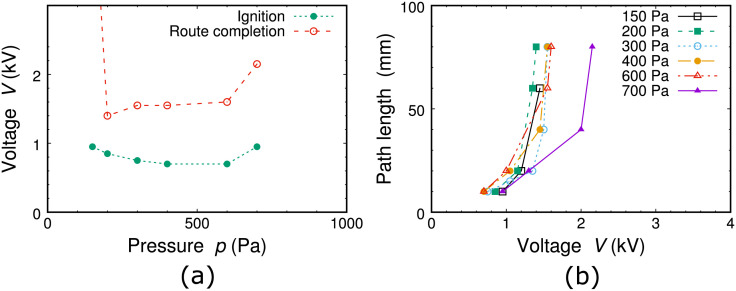
(a) Observed peak discharge voltages for plasma ignition and route completion as a function of discharge gas pressure in case of pattern B depicted in Fig 1 and S1 Appendix. (b) Path-length increments as a function of discharge voltage at various gas pressures in case of pattern B depicted in Fig 1 and S1 Appendix.

In Fig 5(a), in which ignition and route-completed voltages are plotted as a function of Ar gas pressure, we cannot find any sharp minimum property [7], and the possible operation pressure ranges approximately by one order of magnitude. This is attributed to the above model based on the reports about dielectric barrier discharges [21, 22], unlike the case close to pure glow discharges [9]. Fig 5(b) shows evolutions of the route length along the plasma emission channels, and with slight slope changes of the emission lengths, similar completion of route findings is observed at every gas pressure. These facts indicate that the wide operational range is due to partial properties of dielectric barrier discharges, meaning that charged particles accumulated by the previous discharge pulse work effectively to evolute the plasma channels.

All the signals plotted in Fig 5 are shown as photo images in S1 Appendix.

### Experiments: Trends of charge accumulation along route

To perform direct observation of charge accumulation on dielectric surface, we measured the electric potential on one floating electrode *V*_f_ in the middle region along the plasma channel, as shown in Fig 4. Unlike other electrodes, the top of this electrode was installed on the same surface as the surrounding dielectric (in Fig 1). Although the detected signals are not completely the same as that of potential by the accumulated charges on the dielectric surface, it approximates the temporal behavior of the dielectric surface potential.

At the initial phase in which the found route was so short that the plasma emission was far from the floating electrode, the *V*_f_ signal was almost constant as zero. When the front of the emission reached the tip of the electrode, the signal drastically varies as a large swing in the level of several tens of V. This large swing of the potential directly reflects accumulated positive and/or negative discharges at the end of the plasma channel. This charge accumulation takes place on floating capacitance between the dielectric surface and the electrode poles in the equivalent circuit. Floating capacitance always work more or less in experiments of dielectric barrier discharges, and in our case, its effects are getting more apparent as the channel front approaches to the electrode. That is, plasma supplies charges accumulated on the dielectric surface along a channel, and the channel front is connected to the goal electrode by floating capacitance and, in addition, slight charge flows directly in space as Townsend discharge [7], both of which contribute to selection of proper path at a branch. At the final stage in which the floating electrode was immersed in the plasma channel whose one end was on the ground electrode, with the discharge including a direct-current component as a portion, the *V*_f_ signal shows regular alternative changes between relatively large positive and negative potentials. These alternative swings of potentials are due to positive and negative charging synchronized with the alternative discharge voltage. From this measurement, one can confirm the fact that the dielectric surface attached to the plasma was certainly charged up, and the dielectric-barrier-discharge component worked effectively in this route finding phenomenon.

**Fig 6:**
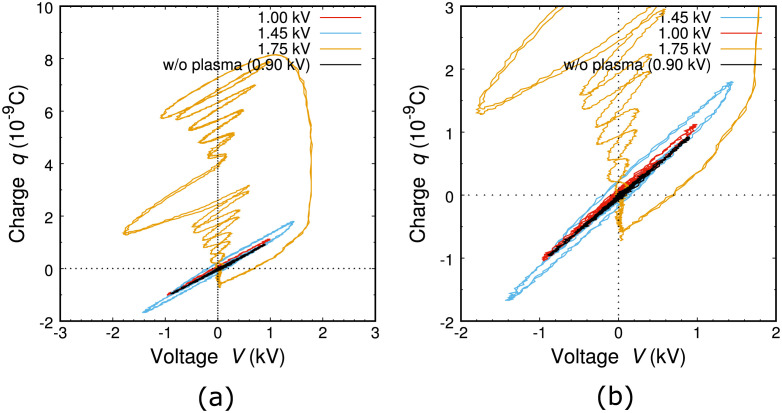
(a) Lissajous curves, or charge-voltage diagrams derived from time-evolution signals in case of pattern A in Fig 4. (b) Enlarged view of (a).

A more closer look of signals in the time evolutions enables us to quantify the accumulated charges stored on the surface. By making a connection between discharge voltage and accumulated charge *q*, integrated from current signals in time, charge-voltage diagrams were derived from time-evolution signals in Fig 4; these diagrams are Lissajous curves that are deduced from two synchronous cycle signals, displayed in Fig 6. Since two cycles are included in Fig 4 and the diagrams are almost on the same curve, such alternative charging through discharges is repetitive. When the peak voltage is in the lower level (< 1.5 kV), *q* increased as the voltage was raised, which corresponds to the route extension from the entry to the goal. Another point we can find is that the summed-up *q* was balanced between the positive and negative amounts, which are typical cases for dielectric barrier discharges. When the peak voltage was in the high level (> 1.7 kV), as we have already noted in one of the current signals in Fig 4, large positive spikes emerged and pulsed direct-current discharges were dominant, leading to more intense emissions from plasma in Fig 3.

The area within a closed Lissajous curve is equal to the power consumption or heat injection to the system. The calculation reveals that the input power ranged from 0.1 to 3.0 W. This is equal to the input quantity of heat in the thermodynamical point of view.

### RL model analysis: Spatial transport of rewards and route finding processes

**Fig 7:**
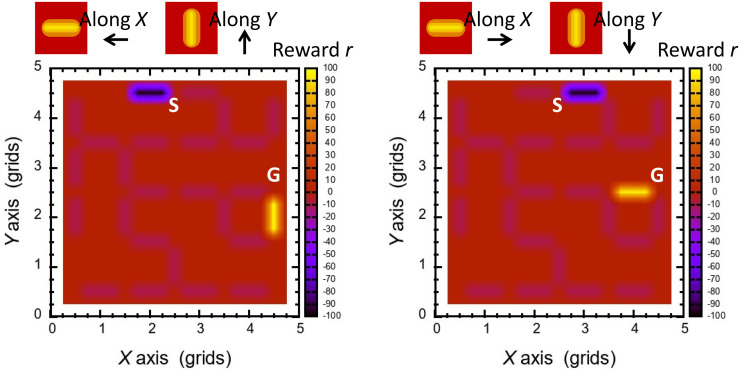
Spatial profiles of initial reward *r* for RL model in configuration of pattern A. The adjacent path from the entry and that to the goal are −100 and +100, while the other edges have slight negative values (−10).

**Fig 8:**
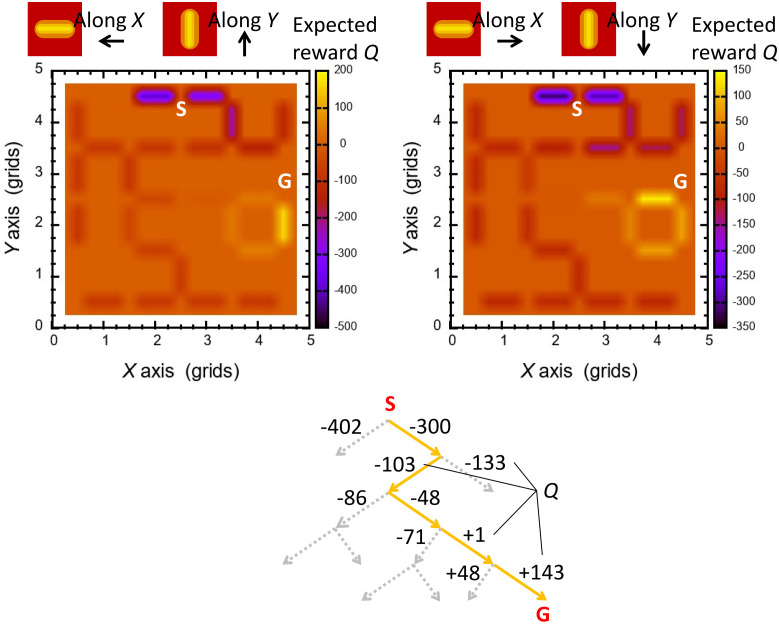
Spatial profiles of expected discounted reward *Q* calculated in RL model in configuration of pattern A, and result of a route finding by selecting larger *Q* at every branch.

Using the patterns copied from the spatial node and edge configurations in the maze targeted in the experiments, we performed simple calculations of route findings based on the RL model based on Eq (2). Figs 7 and 8 represent initial setting of *r* on the edges and calculated *Q* in the model. For each pattern with a different position of the goal node, both increment and decrement effects on *Q* diverge from the entry and converge on the goal node, respectively.

After saturation of a *Q* profile, an agent starts from the entry node and proceeds by choosing an edge for the maximum increase of *Q* in the positive polarity. Then, in both spatial patterns, the agent successfully finds the shortest route whose end was the goal node, as shown in Fig 8 in configuration of pattern A. Both polarities in *r* and *Q* are valid and effective for the guide of the agent; negative *Q* expels the agent, whereas positive *Q* attracts it. Around the starting node the edges has almost the minimum quantities of *Q* with large numerals in the negative sign, and the agent is expelled from such low-level states. The agent aims at selecting a path with larger *Q* to the positive sign, yielding that its trace coincides with increment of *Q* to the goal.

**Fig 9:**
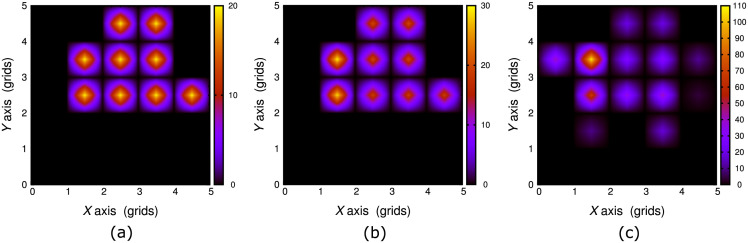
Counts of visits among 20 agents at each node for various initial setting of *r* in configuration of pattern A. *r* = −10 for major edges except regions near the entry and the goal, as indicated below. (a) Reward around the entry: *r*_entry_ = −100, around the goal: *r*_goal_ = +100. (b) Reward around the entry: *r*_entry_ = −11, around the goal: *r*_goal_ = −7. (c) Reward around the entry: *r*_entry_ = −11, around the goal: *r*_goal_ = −9.

We examined what effect(s) will cause failures in route findings. Fig 9 displays profiles of visiting nodes in several cases of *r* around the entry and the goal node. When *r* around these nodes are distinguishable from that on major and general edges, route findings are more successful, then *r* on the background field on the way may work as error sources or obstacles. This fact resembles possible physical cases in which charged density profiles are flat and no further plasma generation is expected.

## Discussion

### Comparison between plasma generation and RL model: From formula structure

As shown and described in the experimental results, charge accumulation on the dielectric walls was a key factor when the plasma emissions increased its length. The source of charged particles is plasma itself generated in the microchannels. The transport of plasma charged particles with density *n* is given, in the particle balance equation, as:

$$\begin{array}{c} \frac{\text{d}n_{c}}{\text{d}t}=-\nabla \cdot (\mu_{s}En_{c}-D_{c}\nabla n_{c})+S_{c}, \end{array}$$ (4)

where *c* represents species of particles, which are charged positively (like ions, symbolized as ‘+’) or negatively (like electrons, as ‘−’), and

$$\begin{array}{c} \nabla \cdot (\varepsilon \nabla \phi )=-e(n_{+}-n_{-}), \end{array}$$ (5)

with *E* = −∇*ϕ*. *ϕ* is the electric potential whose boundary conditions are applied as the discharge voltage and the grounded level. $S_{c}$ is the source of plasma particles, mainly via ionization, *μ* the drift mobility, and *D* the diffusion coefficient. In the bulk region inside plasma, which is the very long part along the channel in our plasma, *n*_+_ ∼ *n*_−_, then electric field *E* is almost constant and relatively small in the bulk, while strong *E* field and its gradient are present on the end regions.

For the following discussion, it is useful to rewrite Eq (2) as:

$$\begin{array}{c} \frac{Q_{i}\left(s_{j},a_{j}\left(s_{j}\right)\right)-Q_{i-1}\left(s_{j},a_{j}\left(s_{j}\right)\right)}{\delta t}\\ =-\frac{\alpha }{\delta t}[Q_{i-1}\left(s_{j},a_{j}\left(s_{j}\right)\right)-\gamma \;\underset{a_{j+1}\left(s_{j+1}\right)}{\text{random}}\;Q_{i-1}\left(s_{j+1},a_{j+1}\left(s_{j+1}\right)\right)]+\frac{\alpha }{\delta t}r_{j}. \end{array}$$ (6)

In this form, it is clear that the left hand side of Eq (6) indicates the finite difference method in solving differential equations numerically as well as an update of *Q* in every *δt* step. Eqs (4) and (6) shares several similar parts, with certain uncommon points; in both cases, source terms exist ($S_{c}$ in Eq (4) and (*α*/*δt*)*r*_*j*_ in Eq (6)), and the diffusion term (*D*_*c*_∇*n*_*c*_) in Eq (4) and the random spatial-shift term (*γ* random *Q*_*i*−1_ in Eq (6)) form spatial profiles of charged particles and *Q*. A different point is that, in Eq (4), spatial divergence is dominant, whereas the learning rate (*α*) is the alternative factor in Eq (6). This partial similarity with different factor(s) is a key in analogous resemblance between two systems handled in this study. If one made them closer analogously, a further modified Q-learning model might be possible which mimics a given physical/chemical/biological system; for instance, *α* can be set not as a constant but as a function of spatial divergence to approach to some plasma systems.

In this way, keeping on the similar RL protocol and calculation setup, RL-based methods can be developed as a physics-informed model for other purposes by modifying and/or adding some components in the model; in our case, we replace the element *max* by the function *random* to imitate particle diffusion as a first step. Recently, several studies have reported linkages between RL models and complex optimization problems in diverse fields of physics [18]. Similar information flow may exist in other systems as well as in this plasma-channel networks. Thus, reinforcement-learning models have a practical potential to work as a generalized approximator which is appliable for other physical/chemical/biological systems. This study will also contribute to recent progresses in such research activities.

From this comparison between plasma experiments and RL models, we can deduce another perspective on the future potentials of plasma maze-solving technology. From Fig 4, the time evolution of four bipolar pulses were completed within 100 *μ*s, which can be estimated as a rough elapsed time of maze-solving completion. Although enlargement of maze configurations make this time longer, it is so rapid in comparison with typical calculation time of RL models in a conventional personal computer (PC). If we design a compact plasma cell and an interface board with PC, we can develop an analog/digital hybrid device for rapid calculation. As an industrial application, a solver for logistic routing problem [25] will be designable after sufficient model reconstruction.

### Entropy comprehension in thermodynamics and information theory

In this study, we have taken Shannon entropy *H* into account for quantifying a route converging process. *H* is well matched to the research activities in information theory, including RL models so far [18, 27, 28]. On the other hand, plasma is a kind of thermodynamical processes in which input of heat and output of work exist. Thus, it is worth highlighting that *H* estimated in this study can be linked to Boltzmann-Gibbs entropy *S*, as discussed in the following. It is known that, in the thermodynamical point of view, *H* is equivalent to *S* [29–34], where *S* in the total system never decreases as predicted in the Second Law of thermodynamics [35, 36].

In our experiments, as the discharge voltage was raised, the lengths of the plasma channel increased. This configurational change corresponds to increased volume *V* with a constant charged-particle density *n*. In a simple thermodynamical model, when the amount of substance *N* is changed by a factor of λ at a constant gas pressure and temperature, *V*, energy *U*, and *S* in the system have extensivity like: λ*U*(*S*, *V*, *N*) = *U*(λ*S*, λ*V*, λ*N*) and λ*S*(*U*, *V*, *N*) = *S*(λ*U*, λ*V*, λ*N*) [35]. We carefully note that this is valid when constituent particles are in equilibrium over the whole volume, and our experiments in which the gas pressure was at several hundreds of Pa are the cases within this criteria; if the pressure increases the level close to the atmospheric pressure [37], viscous forces in gas flows are dominant over particle diffusions and may break this principle. Within the criteria of homogeneous low pressure throughout the system, the extensivity described here means that, for constant *n*, change of *V* is a reversible process without irreversible parts since the extensivity of *S* is valid due to the reversibility. In fact, when we change the discharge voltage very slowly, the variation of the plasma emission length was reversible in the experiments.

This property verifies firm linkage between the observed *H* and the thermodynamical entropy *S*; here, we note again that, in information theory, *H* is broadly defined using probability distributions in which the summation of probabilities is unity, somewhat apart from thermodynamics. In the experiments, *H* decreased, unlike cases of reversible (with constant *S*) or irreversible processes (with increasing *S*). This experimental fact is attributed to gradual system transition from plasma free expansion to plasma restricted growth (with route findings). In Fig 3, thermodynamically, longer the plasma length is, larger *S* should be linearly. However, in comparison with the case of free expansion of plasma which would fill all the microchannels equally at last, *S* in the restricted growth should be smaller as if *S* *decreased*. In other words, the system in the initial phase of plasma ignition with larger *H* (or *S*) is different from that in the final stage with successful route findings and smaller *H* (or *S*).

This reasonable linkage between *H* and *S* is also meaningful as an evidence of plasma capabilities for maze-route findings as well as an example of physics-informed RL models. Maze solving seems to be *intelligent*, despite of plasma appearances without high-level nonlinearity or complexity like human brains. However, in a closer look at plasma, its feasible capability is based on interactions among huge number of particles. Most of them might be random in the thermal equilibrium, but a few fraction of them may work as a nonlinear electronic device in non-equilibrium components in the total multi-particle system. Plasma used here is completely a thermodynamical open system, with a stationary state of constant outlook. Such a system is frequently robust for work output, like biological livings [38], and this maze-solving phenomenon by plasma channels can be listed in examples of functional open systems.

## Conclusion

Maze-solving by plasma emissions was successful in experiments using dielectric microchannels whose synthesized structure imitates a maze configuration, and a RL model reconfigured the similar route-finding processes. Plasma was generated in AC surface dielectric barrier discharges with a small portion of DC volume mode, where charge particles are indispensable for progressive route extension. The RL model used here was typical, except that initial rewards were set negatively around the entry and positively the goal of the maze, and route findings were also successful. The rewards were distributed through the maze, which was roughly similar to the charged-particle transport in the experiments. Shannon entropy calculated from route-finding processes well represented route converging, and consistent with thermodynamical aspects, which is conclusive for consistency between experiments and RL model calculations.

## Supporting information

S1 Appendix Supplementary document. pdf of supplementary document referred to in the main text. They include raw data and essential parts of numerical codes without dataset input/output parts that are specific for each computational device. The raw data listed here are before data handling such as sampling and integration of data points. The part of the numerical code shown here is sufficient for data reproduction, with parameters listed in the main document.
(PDF)
