# Supplementary material for: Maze-solving in a plasma system based on functional analogies to reinforcement-learning model
Source: PLoS One. 2024 Apr 10;19(4):e0300842. doi: 10.1371/journal.pone.0300842 (PMC11006191; doi:10.1371/journal.pone.0300842)
Supplement: S1 Appendix — pdf of supplementary document referred to in the main text. They include raw data and essential parts of numerical codes without dataset input/output parts that are specific for each computational device. The raw data listed here are before data handling such as sampling and integration of data points. The part of the numerical code shown here is sufficient for data reproduction, with parameters listed in the main document. (PDF) [file pone.0300842.s001.pdf]

Supplementary Document for: Maze-solving in a plasma system based on functional analogies to reinforcement-learning model

Osamu Sakai[1,2*], Toshifusa Karasaki[1], Tsuyohito Ito[3], Tomoyuki Murakami[4], Manabu Tanaka[5], Makoto Kambara[6], Satoshi Hirayama[1,2]

1 Department of Electronic Systems Engineering, The University of Shiga Prefecture, Hikone, Shiga, Japan
2 Regional ICT Research Center for Human, Industry and Future, The University of Shiga Prefecture, Hikone, Shiga, Japan
3 Department of Advanced Materials Science, The University of Tokyo, Kashiwa, Chiba, Japan
4 Department of Systems Design Engineering, Seikei University, Musashino, Tokyo, Japan
5 Department of Chemical Engineering, Kyushu University, Fukuoka, Japan
6 Department of Materials and Manufacturing Science, Osaka University, Suita, Osaka, Japan

* sakai.o@e.usp.ac.jp

Abstract

In this supplementary document, we supply base datasets and materials for the manuscript description in this study. They include raw data and essential parts of numerical codes. The raw data are before data handling such as sampling and integration of data points. The part of the numerical code shown here is sufficient for data reproduction, with parameters listed in the main document.

Details of experimental design and results

Time evolutions of discharge signals

The datasets of raw signals collected in experiments of pattern A are shown here (Fig A1). In the manuscript, we mainly use time evolutions in the configuration of pattern A for electrical measurements. For instance, charge q shown in Fig 6 is derived from the time evolution of the signal of current I by integrating it in time. We note that, to extract net discharge current, we remove the component of displacement current from the raw signals which is available in case without plasma generation.

Photo images of route-finding process

Pattern B (shown in Fig A2), in which goal location was moved from that in pattern A, was used to obtain dependences of discharge parameters, and all visible photo images in pattern B are shown in Fig A3.

In these cases, the images of plasma emission reveal that route finding is also successful, although plasma channel emissions show differences depending on gas

pressure and discharge voltage. More specifically, at lower pressures (e.g., at 150 Pa and 200 Pa), more branches are visible and final route from the entry to the goal is less clear. At higher pressures (e.g., at 400-700 Pa), the final route was intensified by DC currents directly flowing between the two electrodes corresponding to the entry and the goal, which implies more converged solution for maze.

Details of Reinforcement model

Framework and coding in Python

The framework of our RL model is listed in algorithm below. This is a simple and typical one for maze solving.

Algorithm: computational code for RL model

```
1: Set parameters and initialize variables
2: Input spatial profiles of rewards
3: Update Q iteratively
4: Find path from initial node as Q becomes maximal in positive polarity
5: Output path
```

The core part of the RL model in Fig 2 is for updates of Q values, represented by Eq (2). It is listed in the following in the format of Python [1], and unlike the usual code in which the next, next position is selected to fulfill maximum Q gain, we set Q diffusion through random microscopic actions. The entire code is available from the corresponding author upon reasonable request.

List: partial code for Q undates in RL model

```
for i in range(10000): #Iterations up to 10000
  p_state = np.random.randint(0,numNode)
  #Select tentative position at random
  n_actions = [] #Set list for candidates of next position
  for j in range(numNode): #Iterations up to node number (numNode)
    if abs(reward[p_state,j]) >= 1: #If reward is nonzero
      n_actions.append(j)
      #Add it to list of candidates from current position
  n_state = np.random.choice(n_actions) #Select randomly from candidates
  nn_actions = [] \#Set list for candidates of next, next position
  for j in range(numNode): #Iterations up to node number (numNode)
    if abs(reward[n_state,j]) >= 1: #If reward is nonzero
      nn_actions.append(j)
      #Add it to list of candidates from next position
  nn_state = np.random.choice(nn_actions)
```

```
#Select randomly from candidates
Q[p_state,n_state]
  = (1-alpha)*Q[p_state,n_state]
  +alpha*(reward[p_state,n_state]+gamma*Q[n_state,nn_state]) #Update Q
```

References

1. Python Software Foundation. https://www.python.org/

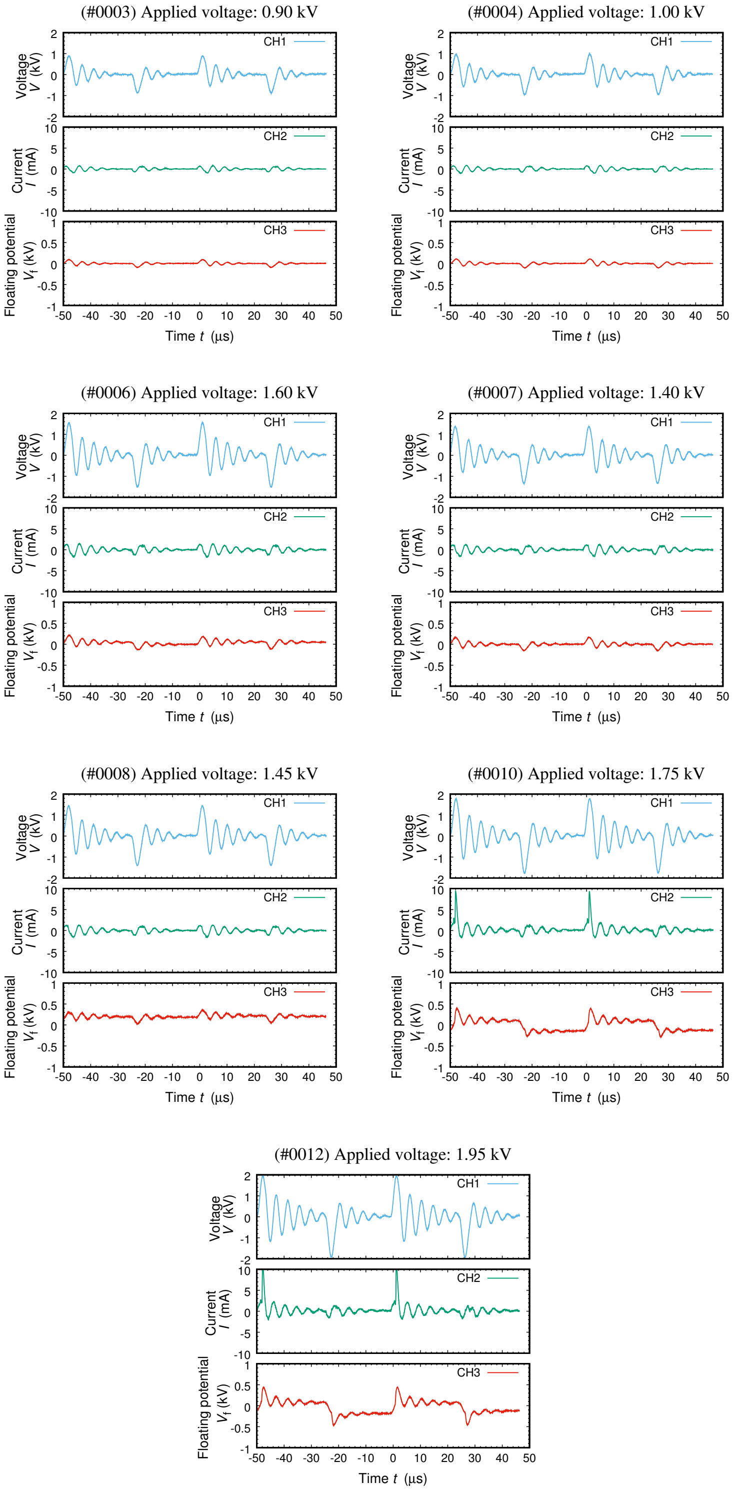

Fig A1. Time evolutions of raw signals in case of pattern A. Parameters shown as inset numerals in voltage correspond to the bipolar peak voltage, with Ar gas pressure 300 Pa.

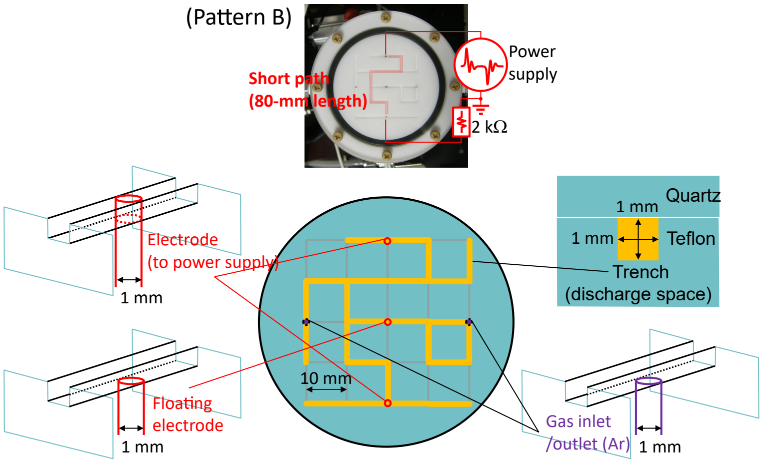

Fig A2. Schematic view of pattern B used in experiments for investigating parameter dependences.

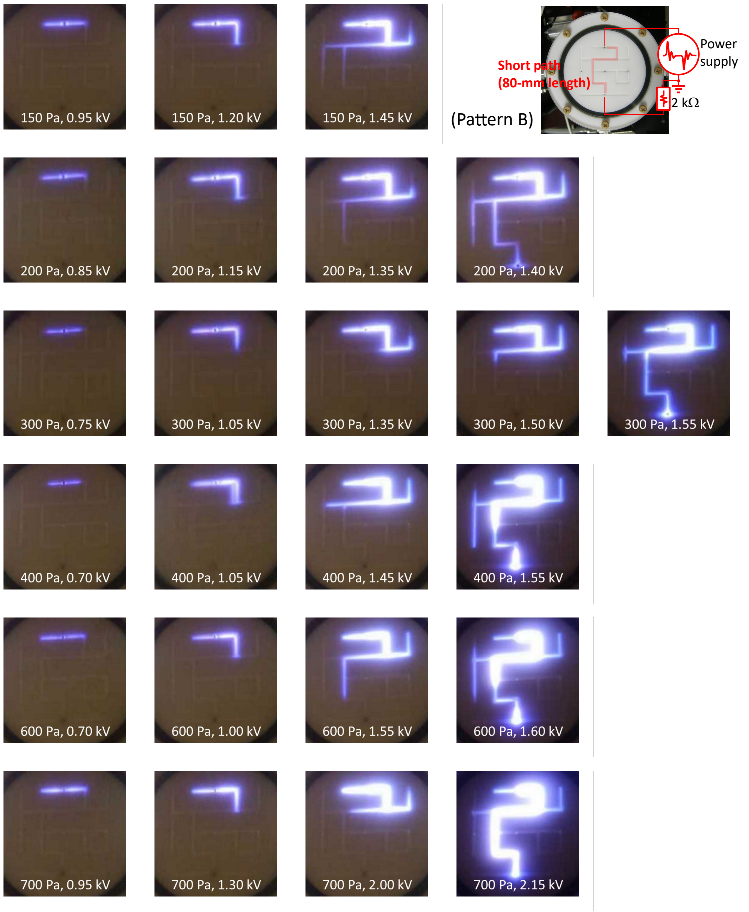

Fig A3. All visible images of plasma channels in pattern B at different gas pressures with varying discharge voltage.
